# A Rare Case of Arnold Chiari Malformation Type 1 Presenting With Features of Catatonia

**DOI:** 10.7759/cureus.65321

**Published:** 2024-07-24

**Authors:** Janani Duraisamy, Ramya Rachel Jetty, Sivabackiya C, Aruna Kaki, Arul Saravanan R

**Affiliations:** 1 Psychiatry, Sri Ramaswamy Memorial (SRM) Medical College Hospital and Research Centre, Chennai, IND; 2 Psychiatry, Sri Ramaswamy Memorial (SRM) Medical College Hospital and Research Centre, Kattankulathur, IND; 3 Psychiatry, Sri Ramaswamy Memorial (SRM) Medical College Hospital and Research Centre, Institute of Science and Technology, Chengalpattu, IND; 4 Psychiatry, Sri Ramaswamy Memorial (SRM) Medical College Hospital and Research Centre, Institute of Science and Technology, Chennai, IND

**Keywords:** postpartum psychosis, liaison, catatonia, headache, hydrocephalus, arnold chiari malformation

## Abstract

The Arnold Chiari malformation is a congenital neurological condition. It occurs due to a defect in the cerebellum. Our patient is a 19-year-old postpartum female who presented to our ER with headaches, crying spells, reduced interaction, poor self-care, and neglect of her newborn for the past five days. Before the onset of her presenting symptoms, the patient had complained of a severe headache in the back of the head and dizziness. Her baseline investigations were normal. On examination, the patient was noticed to have a fixed gaze, ambiguity, mutism, and rigidity. So, she was diagnosed with catatonia, a differential diagnosis of mental and behavioral disorders associated with pregnancy, childbirth, and puerperium, and was treated with medications appropriately. As her headache showed minimal relief with adequate analgesic measures, neuroimaging was done, which showed Arnold Chiari malformation type I with hydrocephalus. A liaison was made with the neurology team, who confirmed the diagnosis and advised her on the decompression procedure. Her family refused to undergo the procedure. Once she showed minimal improvement in her symptoms, her family members requested her discharge. In our case, the Arnold Chiari malformation type I presented with features of catatonia, unlike the usual reported presentation of depression and anxiety. This case highlights the need for a thorough evaluation of any patient presenting with catatonia.

## Introduction

The Arnold Chiari malformation is a congenital neurological condition that occurs due to a defect in the cerebellum. The cerebellum may or may not get displaced through the subarachnoid space into the cervical spine. Arnold Chiari malformation is of four types: Type I to IV, based on the degree of displacement of the cerebellum and the mid-brain structures. In a type I malformation, only the cerebellar tonsils slip through the foramen magnum into the cervical spine due to reduced posterior fossa volume. In Type II malformation, the cerebellar tonsils, along with mid-brain structures, descend into the spine and may be associated with spina bifida. Type III malformation is the most severe form with cervical encephalocele, and lastly, Type IV malformation is the rarest type with an absent or underdeveloped cerebellum. Type II is generally identified in childhood due to the presence of myelomeningocele, while type III and type IV are found to occur very rarely, with the former having a good to poor prognosis based on the structures involved, while the latter two have the worst prognosis. The main treatment option for this condition is surgical decompression, which may or may not improve the outcome, depending on the severity and duration of the illness.

Arnold Chiari type I malformation occurs in about 0.5-3.5% of the general population, with a male: female gender prevalence of 1:1.3, respectively [1]. The prognosis for this condition is generally positive, especially in patients without preexisting neurological impairments, as they tend to have favorable outcomes [2]. It usually presents as a persistent sub-occipital headache, just like in our patient, with neck discomfort noticed in 80% of patients, which often intensifies due to pressure, for example, while coughing. Patients may also experience dizziness, vertigo, constant fatigue, and visual and gait disturbances. Few studies have reported cognitive deficits such as impaired attention, memory, visuospatial performance, etc., associated with this malformation. Psychiatric comorbidities like mood disorders, anxiety spectrum disorders, psychosis, and, rarely, attention deficit hyperactivity disorder (ADHD) have also been reported with this condition [3]. Ongoing recent research is in the midst of exploring the non-motor functions of the cerebellum (limbic cerebellum in posterior cerebellum) like executive function, linguistic skills, affect regulation, and visuospatial processing using functional MRI (fMRI), in particular, its influence on cognitive functioning and emotional processing [4,5].

The prevalence of postpartum psychosis is 1-3 per 1000 births, according to the data collected. The onset is usually abrupt (within hours), with the episodes commonly presenting between 3 and 10 days after the onset of the postpartum period. The early warning symptoms of postpartum psychosis include sleep disturbance, low mood, anxiety, irritability, elation, confusion, and, at times, even disorganization in behavior. In an Indian study, it was found that about 20% of the 200 women suffering from postpartum psychosis present as catatonia, with the most prevalent symptoms being mutism, withdrawal, and negativism [6].

Catatonia is a neuropsychiatric disorder characterized by motor, behavioral, and emotional symptoms of immobility, mutism, stupor, and agitation. Organic catatonia is a subtype of catatonia that arises secondary to underlying medical or neurological conditions, such as metabolic imbalances, infections, or brain injuries. The researchers emphasize the importance of promptly identifying and addressing these underlying organic causes to ensure appropriate and effective treatment of organic catatonia. 

## Case presentation

**Figure 1:**
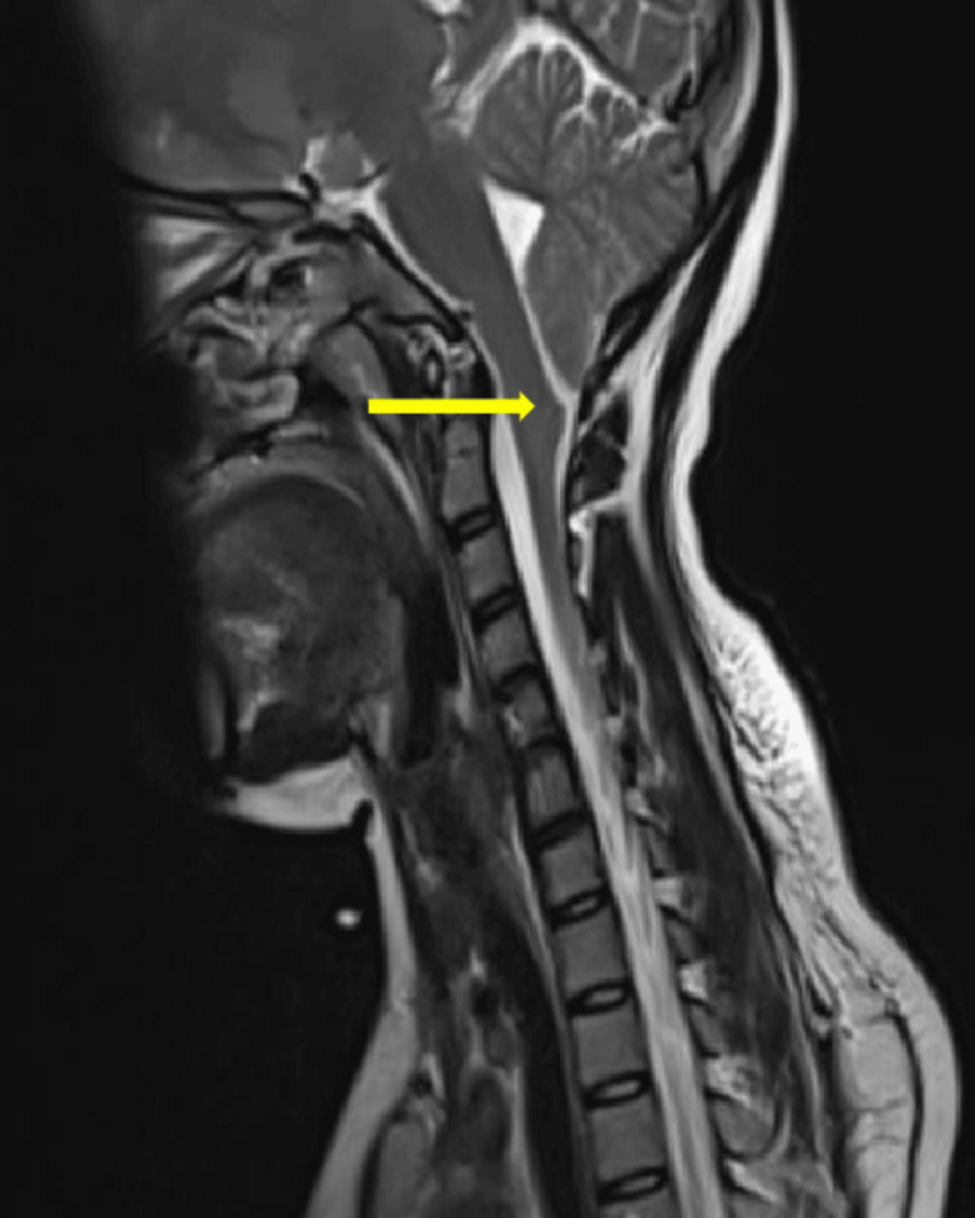
MRI spine T2 Sagittal section: showing Arnold Chiari malformation type 1 (arrow shows the displaced cerebellar tonsils)

**Figure 2:**
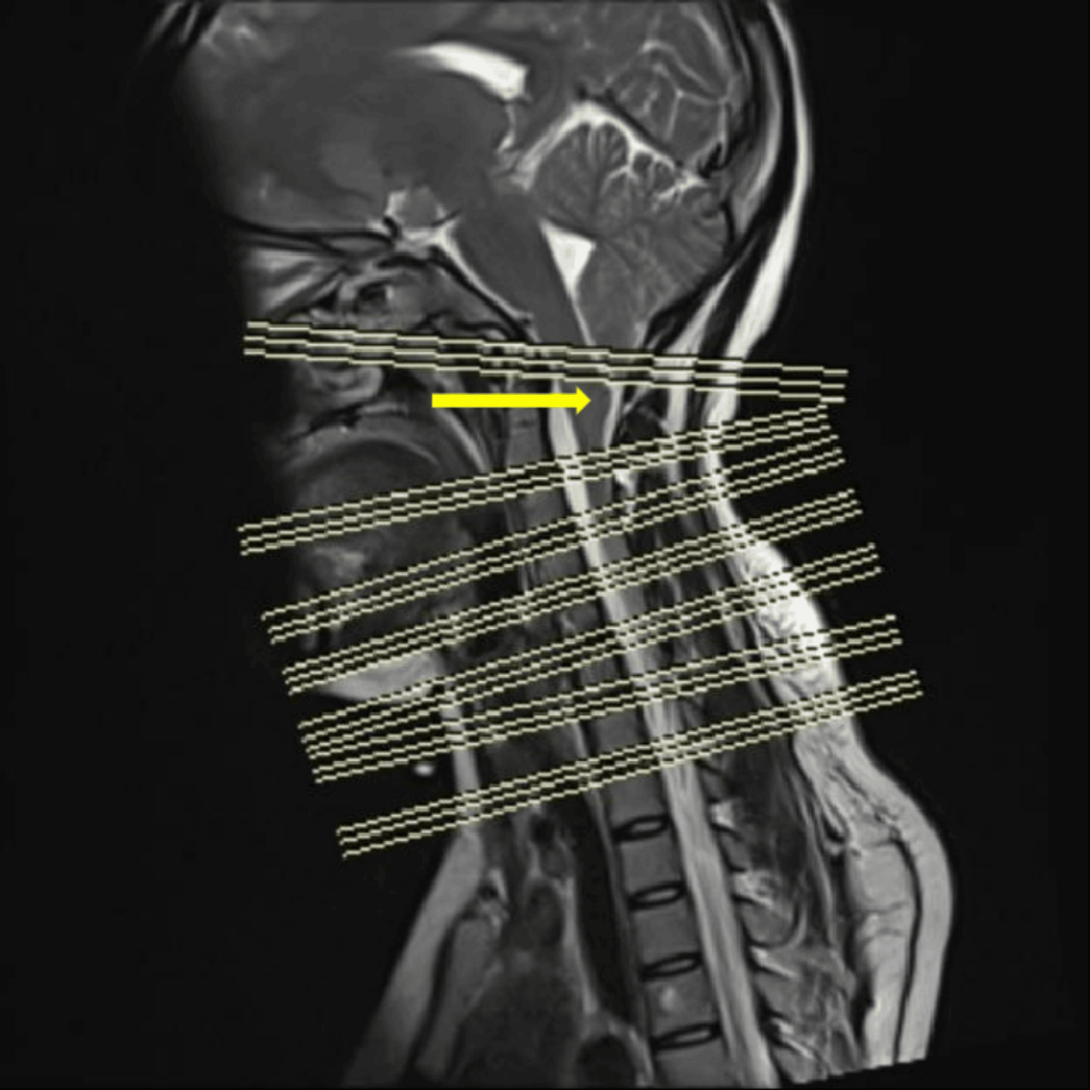
MRI spine T2 sagittal section cerebellar tonsils were found to be slightly displaced below the Mcrae line, suggesting Arnold Chiari malformation type I (Triple line demarcation is done here to represent the inter-vertebral disc space, with the first line being the Mcrae line)

**Figure 3:**
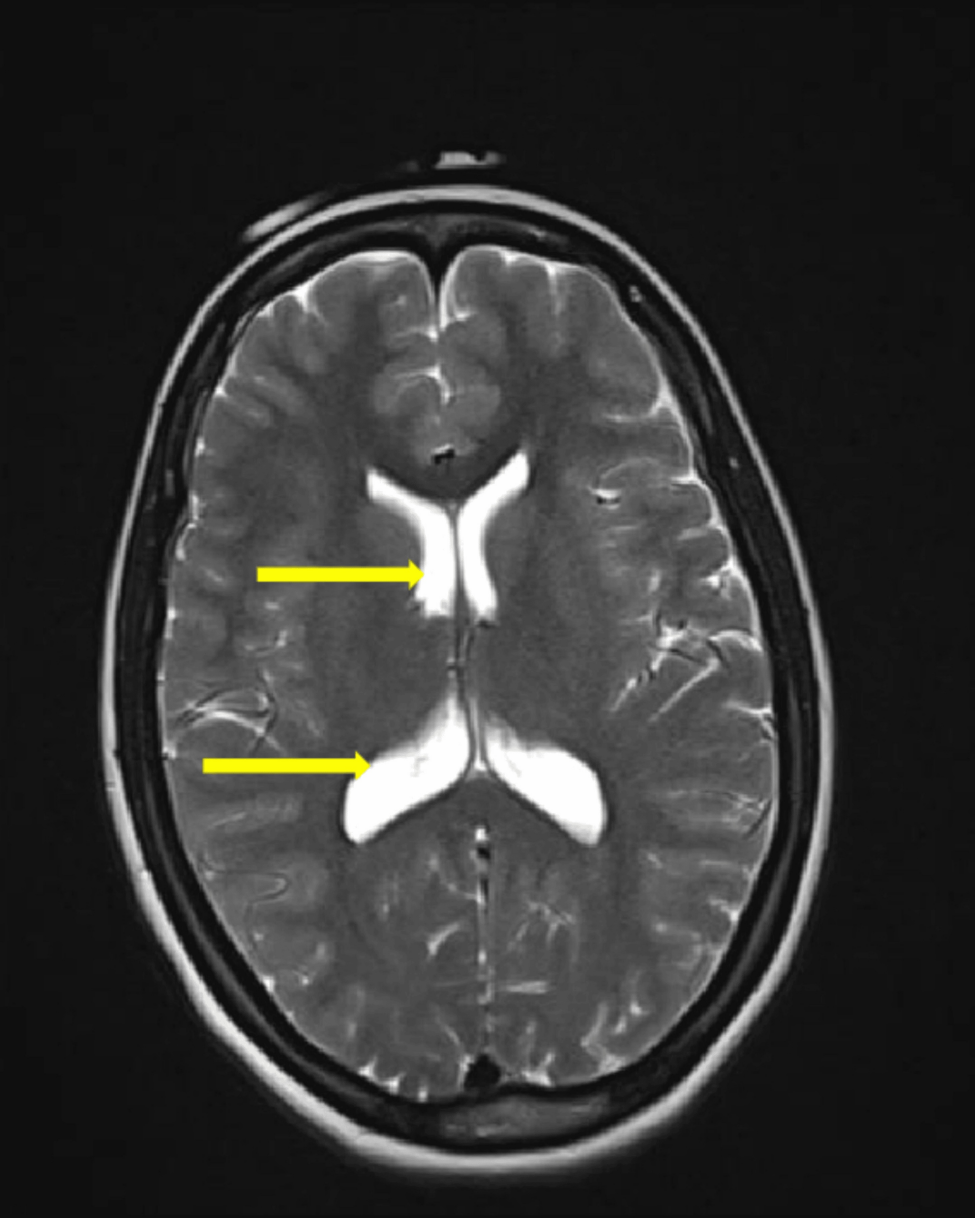
MRI brain T2 axial section shows hydrocephalus (arrows show enlarged ventricles)

A 19-year-old woman who gave birth a month ago presented with a progressively worsening headache, which was exacerbated by coughing and bending down. Initially, she managed her symptoms with over-the-counter analgesics, but her condition continued to deteriorate. Her daily routines, including caring for her child and maintaining household chores, were significantly affected. Despite her mother’s assistance, her sleep progressively reduced, and she became increasingly irritable, snapping at her crying child. She gradually withdrew from interactions and was mostly bedridden, crying and staring at the ceiling. Later, she avoided bathing or using the restroom for almost two days. Her husband and mother, concerned about her deteriorating condition, brought her to our emergency room. On examination, she exhibited a fixed gaze, mutism, and rigidity. Her Bush-Francis Catatonia rating scale score was six, leading to a diagnosis of 6A4Z. Unspecified catatonia with a differential diagnosis of 6E2Z Mental and behavioral disorders are associated with pregnancy, childbirth, and the puerperium, according to the International Classification of Diseases-11 (ICD-11). Since our patient's symptoms started four weeks after giving birth, we considered both postpartum psychosis and catatonia as possible diagnoses. Routine blood investigations, including the complete blood count, renal function test, liver function test, thyroid function test, serum electrolytes, and urine routine, were done and found to be within normal limits. Her general neurological examination was also normal. She was treated with lorazepam 2 mg three times a day orally, which was gradually tapered to twice daily doses as her complaints showed slight improvement. Additionally, Olanzapine 5 mg at night was started orally to address her expressed fearfulness and auditory hallucinations, which developed later. Despite these measures, her headache showed minimal improvement, prompting an MRI of the brain and spine, which revealed Arnold Chiari's malformation type I with hydrocephalus (Figures 1-3).

A liaison was made with the neurologist, who then confirmed the diagnosis and recommended decompression surgery. But, her family refused the procedure for personal reasons despite thorough psychoeducation. Consequently, her diagnosis was revised to secondary catatonia syndrome, as per ICD-11. With medication, she showed minimal improvement in symptoms related to functioning, personal hygiene, and hallucinations, although her headache persisted. Her Bush-Francis Catatonia Rating Scale score at this time was four. At the family's request, the patient was ultimately discharged and could not be followed up on subsequently.

## Discussion

Catatonia, the presentation seen in our patient, was first distinguished by Karl Ludwig Kahlbaum in 1874. It is characterized by a range of psychomotor disturbances, including abnormal movements, immobility, and behavioral abnormalities. Its prevalence is about 5%-18% in inpatient psychiatric units and about 3.3% in neurology or neuropsychiatric tertiary care inpatient units [7-9]. A recent study by Rogers et al., utilizing electronic health records, showed an increasing incidence of catatonia [10]. Despite its high prevalence, catatonia is often widely underdiagnosed.

Arnold Chiari Malformation Type I, diagnosed in our patient, often appears in adulthood with persistent headaches [11]. It can also cause neuropsychiatric symptoms like depression, anxiety, or psychosis [3]. These psychiatric issues are due to the malformation's impact on neurotransmitter levels and the pressure it puts on the brainstem, spinal cord, nerves, and blood vessels. For instance, patients with Arnold Chiari malformation type I may exhibit a range of neuropsychiatric symptoms, including insomnia, agitation, and speech difficulties, which can be resistant to standard antipsychotic treatments [3,12-13]. In another report, a middle-aged woman with depressive and suicidal tendencies initially diagnosed with major depressive disorder was later found to have Chiari malformation type 1 and hydrocephalus by brain MRI. She showed partial improvement in her complaints with suitable antidepressants in the initial month of treatment, but in the following months, she showed a progressive decline. Later, she underwent a decompression procedure. It was suggested by this study that in individuals with conditions such as this, depression may be linked to compression of the brainstem and also alterations in serotonin and noradrenaline levels [3,14]. The presence of a link between mood disorders and Arnold-Chiari malformations is debated. Some experts think that the failure of surgery to improve behavior shows there is no direct link [15]. Thus, it remains unclear if surgery will improve our patient's neuropsychiatric symptoms.

## Conclusions

In conclusion, while neuropsychiatric manifestations in Arnold-Chiari Type 1 malformation are infrequently reported, the most common are depression and psychosis. This particular case, involving an underlying Arnold-Chiari Type 1 malformation, presented in the postpartum period with features of catatonia, a presentation not previously encountered in the literature. This unique presentation highlights the need for clinician awareness and a comprehensive approach, including standardized assessments, to ensure earlier diagnosis and appropriate management.
